# MSGM: a multi-scale spatiotemporal graph Mamba for EEG emotion recognition

**DOI:** 10.3389/fnins.2026.1665145

**Published:** 2026-02-05

**Authors:** Hanwen Liu, Yifeng Gong, Zuwei Yan, Zeheng Zhuang, Jiaxuan Lu

**Affiliations:** 1 The School of Electronics and Communication Engineering, Sun Yat-sen University, Shenzhen, China; 2 The College of Communication Engineering, Jilin University, Changchun, China; 3 The School of Pharmaceutical Sciences (Shenzhen), Sun Yat-sen University, Shenzhen, China; 4 Shanghai Artificial Intelligence Laboratory, Shanghai, China

**Keywords:** electroencephalogram (EEG), emotion recognition, graph neural networks, Mamba, multi-scale

## Abstract

**Introduction:**

Electroencephalography (EEG) based emotion recognition is pivotal for advancing mobile health monitoring and real-time affective interaction. However, current methodologies face a critical trade-off between modeling the complex, multi-scale dynamics of brain activity and maintaining the computational efficiency necessary for edge deployment. Existing approaches often rely on fixed temporal scales and neglect hierarchical spatial connectivity, which limits both classification robustness and scalability in practical settings.

**Methods:**

To address these challenges, we propose the Multi-Scale Spatiotemporal Graph Mamba (MSGM). Specifically, it employs multi-window temporal segmentation to extract relative power spectral density (rPSD) features, mimicking the brain's multi-scale processing to capture both transient emotional fluctuations and sustained mood. Spatially, it constructs bimodal global and local graphs refined by multi-depth Graph Convolutional Networks (GCNs), intuitively modeling hierarchical brain connectivity rather than isolated sensors. These features are synthesized via a token embedding fusion module and processed by a single-layer MSST-Mamba module, which leverages state-space modeling to ensure linear computational complexity, avoiding Transformer latency bottlenecks to facilitate real-time clinical monitoring.

**Results:**

Assessed on the SEED, THU-EP, and FACED datasets under subject-independent protocols, MSGM outperforms baseline approaches, attaining competitive accuracy and F1 scores (e.g., 83.43% accuracy and 85.03% F1 score on SEED). Leveraging a single MSST-Mamba layer, MSGM demonstrates robust generalization and efficiency, achieving millisecond-level inference (151 ms) on the NVIDIA Jetson Xavier NX edge device, confirming its suitability for real-time applications.

**Discussion:**

The capability of MSGM to capture complex spatiotemporal dynamics with low computational overhead highlights its suitability for real-time monitoring and interactive interfaces. By integrating neuroanatomical priors into the selective state-space modeling, the framework effectively maintains spatial intelligence and topological consistency throughout the classification process. This approach not only improves recognition accuracy but also ensures neurophysiologically grounded interpretability. Future research will focus on multimodal integration and further optimization of hierarchical spatial modeling to address the challenges of cross-subject variability. To support research reproducibility, the source code of MSGM will be made available at https://github.com/liuguangyunjizero/MSGM.

## 1 Introduction

Emotion recognition has emerged as a pivotal research frontier with profound implications for human-computer interaction, mental health monitoring, and neuroscientific exploration (Khare and Bajaj, 2021; Cheng et al., 2024; Guo et al., 2022). The capacity to decode emotional states in real-time holds transformative potential for intelligent systems, enhancing user adaptability and enabling clinical applications such as early detection and management of emotional disorders (Jafari et al., 2023; Zhou and Peng, 2024). As these capabilities become increasingly relevant to AI enabled healthcare applications, robust and efficient methods remain essential for bridging theoretical advances with real world deployment constraints (Vafaei and Hosseini, 2025).

Among various physiological signals, electroencephalography (EEG) is a premier modality for emotion recognition due to its non-invasive ability to capture brain activity with high temporal resolution, directly reflecting the neural signatures of emotional processes (Yao et al., 2024). Unlike indirect methods like facial expression analysis or speech intonation, EEG provides immediate access to the brain's dynamic responses, making it ideal for applications demanding precision and responsiveness. However, EEG-based emotion recognition faces significant challenges, including signal susceptibility to noise, spatial heterogeneity across brain regions (Liu et al., 2024), and complex temporal dynamics spanning short-term fluctuations and long-term trends, which fixed-scale approaches often fail to model adequately (Ding et al., 2022).

The methodological landscape of EEG-based emotion recognition has undergone a paradigm shift from manual engineering to automated representation learning (Xiao et al., 2024). While traditional approaches utilized neuroscientific priors to construct interpretable features like wavelet transforms (Murugappan et al., 2010), they remained constrained by labor-intensive processes and limited scalability. Consequently, the field has pivoted toward Deep Learning (DL) architectures to automate this process. Prominent models, including CNNs (Li et al., 2018), RNNs (Soleymani et al., 2016), and Transformers (Vafaei and Hosseini, 2025), have significantly improved performance by integrating features such as relative power spectral density (rPSD) and differential entropy (DE) (Yan et al., 2024). Nevertheless, traditional DL models often treat EEG signals as isolated time series, thereby failing to capture the brain's complex, non-Euclidean spatial topology.

To address these spatial limitations, Graph Neural Networks (GNNs) have emerged as the dominant framework, characterizing EEG channels as nodes within a topological graph. Existing research has explored diverse strategies: Spectral approaches like ChebyNet (Defferrard et al., 2016) employ Chebyshev polynomials to approximate graph Laplacian filters, whereas dynamic methods like DGCNN (Song et al., 2020) optimize adjacency matrices to capture evolving dependencies. Furthermore, to enhance physiological interpretability, models such as RGNN (Zhong et al., 2020) and BiDANN (Li et al., 2021) have incorporated neuroscientific constraints and hemispheric asymmetry, respectively. Despite these advances, most GNN-based methods rely on static or single-scale graphs, which oversimplify the brain's dynamic, hierarchical interactions (Liu et al., 2024) and sacrifice temporal context (Zhang et al., 2019).

Beyond spatial modeling, recent advancements have focused on capturing multi-scale temporal dynamics and achieving spatiotemporal fusion. To handle diverse granularities, architectures such as TimesNet (Wu et al., 2023) transform sequences into multi-scale tensors. Similarly, AMCNN-DGCN (Wang et al., 2020) and Pathformer (Chen et al., 2024) employ multi-scale convolutions and dual attention mechanisms. Cross-modal distillation works, specifically Visual-to-EEG (Zhang et al., 2022), and domain adaptation frameworks like DMATN (Wang et al., 2021), further validate the importance of multi-scale temporal feature extraction (Ding et al., 2023).

Simultaneously, hybrid fusion models have attempted to unify these dimensions. Conformer (Song Y. et al., 2023) combines CNNs and Transformers, while models like SGCN-LSTM (Feng et al., 2022) integrate graph convolutions with recurrent units. Other studies, such as Soleymani et al. (2016)'s work and BiDANN (Li et al., 2021), also excel in fusing EEG features. Notably, recent hybrid optimization strategies have leveraged Continuous Capsule Networks and 3D Cube representations to capture spatial nuances, reporting exceptional accuracies on the DEAP and AMIGOS datasets (Wirawan et al., 2025). However, a critical bottleneck persists. These complex architectures often treat space and time as separate dimensions. More critically, the quadratic complexity inherent in state-of-the-art Transformer-based models renders them computationally prohibitive for real-time deployment on edge devices (Gu and Dao, 2023; Fang et al., 2019).

To resolve the conflict between high-order modeling capability and deployment efficiency, the community has turned to Mamba, also known as State Space Models. By leveraging selective state spaces for linear-time sequence modeling, Mamba offers superior scalability over Transformers, especially for long sequences (Gu and Dao, 2023). Pioneering applications in EEG emotion recognition have shown promise. For instance, Zhou and Peng (2024) utilize a multi-scale Mamba architecture for spatiotemporal fusion, while Global Context Mamba Vision (Wang et al., 2025) combines SSMs with local-global context modeling to enhance efficiency. While these studies validate the utility of SSMs, they lack a unified framework that simultaneously integrates neuroanatomically grounded graphs with the efficient temporal modeling of Mamba. This is a gap this work aims to bridge.

To overcome these limitations, specifically the difficulty in balancing multi-scale spatiotemporal modeling with computational efficiency, we propose the Multi-scale Spatiotemporal Graph Mamba (MSGM) framework. This framework integrates a novel graph-based Mamba structure to model the intricate dynamics of EEG signals comprehensively. Our approach addresses three key aspects.

Firstly, it captures multi-scale temporal dynamics, spanning short-term fluctuations and long-term trends. It uses a multi-window sliding strategy to extract rPSD features from seven frequency bands via the Temporal Multi-scale Feature Extraction module, overcoming the limitations of fixed temporal windows (Ding et al., 2023). Secondly, it models the brain's distributed and hierarchical emotional processing (Zhang et al., 2021) with adaptive global and local graphs. These graphs are constructed using neuroanatomical priors and fused via multi-depth GCNs and token embeddings in the Spatiotemporal Feature Adaptive Fusion modules, directly addressing the shortcomings of single-scale spatial representations (Xue et al., 2022). Thirdly, MSGM ensures edge efficiency with inference times below 151 ms on the NVIDIA Jetson Xavier NX, effectively mitigating the high computational costs that impede real-time deployment .

Our MSGM framework advances EEG-based emotion recognition with the following contributions:

(1) We propose the MSGM network to address subject-independent emotion classification, decoding complex EEG emotional dynamics with high precision.
(2) We introduce the Temporal Multi-scale Feature Extraction, Spatial Multi-scale Prior Information Initialization, and Spatiotemporal Feature Adaptive Fusion modules to enhance modeling of temporal granularity and spatial connectivity.
(3) MSGM delivers superior performance on the SEED (Zheng and Lu, 2015), THU-EP (Hu et al., 2022), and FACED datasets (Chen et al., 2023), surpassing baselines such as DGCNN (Song et al., 2020) in subject-independent settings. Notably, with only a single MSST-Mamba layer, it outperforms leading methods in the field on the same datasets.
(4) Deployed on the NVIDIA Jetson Xavier NX, MSGM delivers real-time inference within 151 ms, enabling efficient performance on resource-constrained edge devices.

## 2 Materials and methods

**Figure 1:**
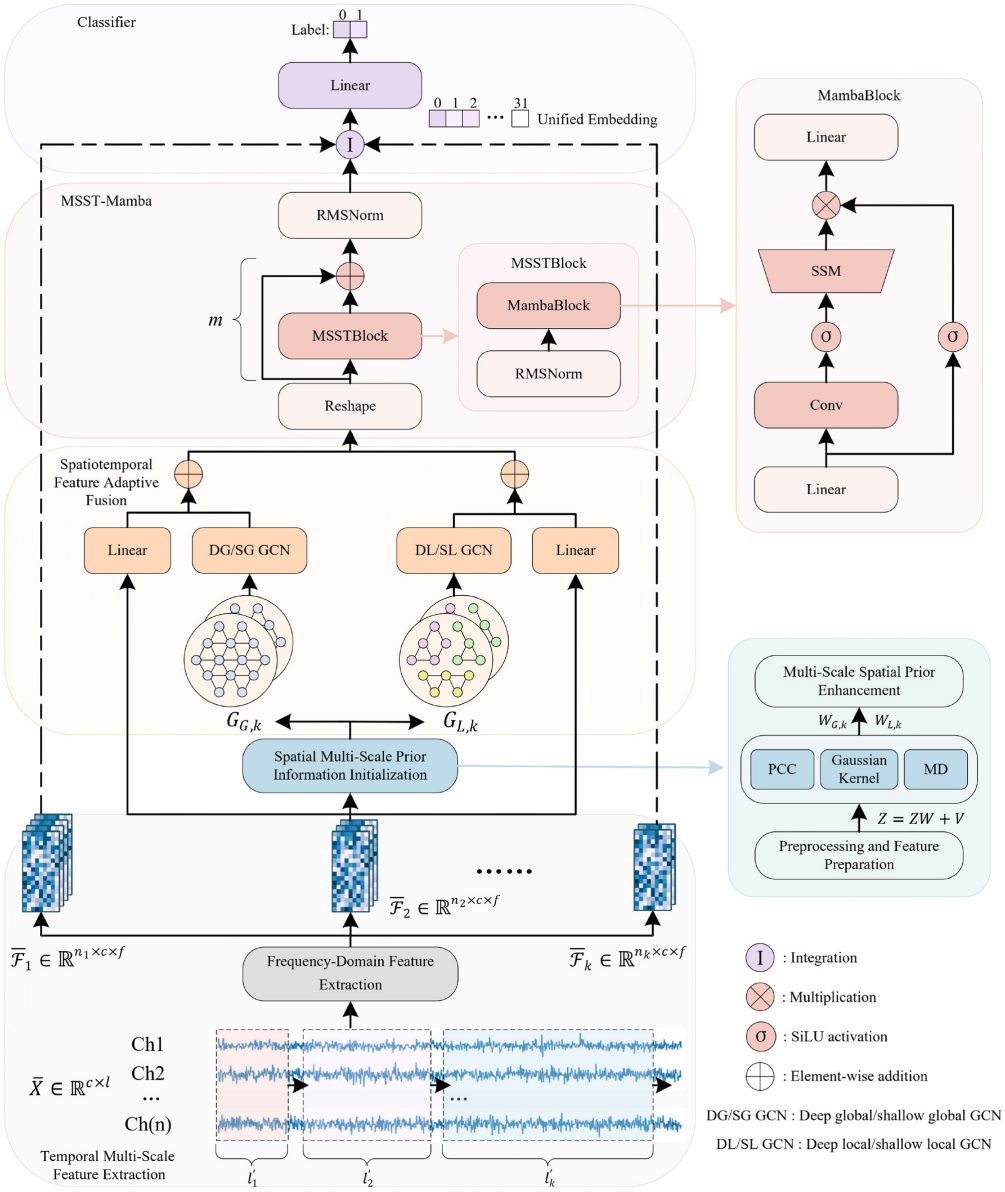
The framework of MSGM. The multi-scale feature tensors from Temporal Multi-Scale Feature Extraction module are used as the input to Spatial Multi-Scale Prior Information Initialization module that will transfer tensors into global graphs and local graphs. Then Spatiotemporal Feature Adaptive Fusion module extract dynamic spatial relationships among EEG channels via GCNs and temporal fusion. The MSST-Mamba block then processes the input tensors, followed by the final Classifier module.

In this section, we present the details of the proposed method, which comprises temporal multi-scale feature extraction, spatial multi-scale prior information initialization, spatiotemporal feature adaptive fusion, MSST-Mamba and classifier. The overall architecture of the proposed method is depicted in Figure 1.

### 2.1 Temporal multi-scale feature extraction

**Figure 2:**
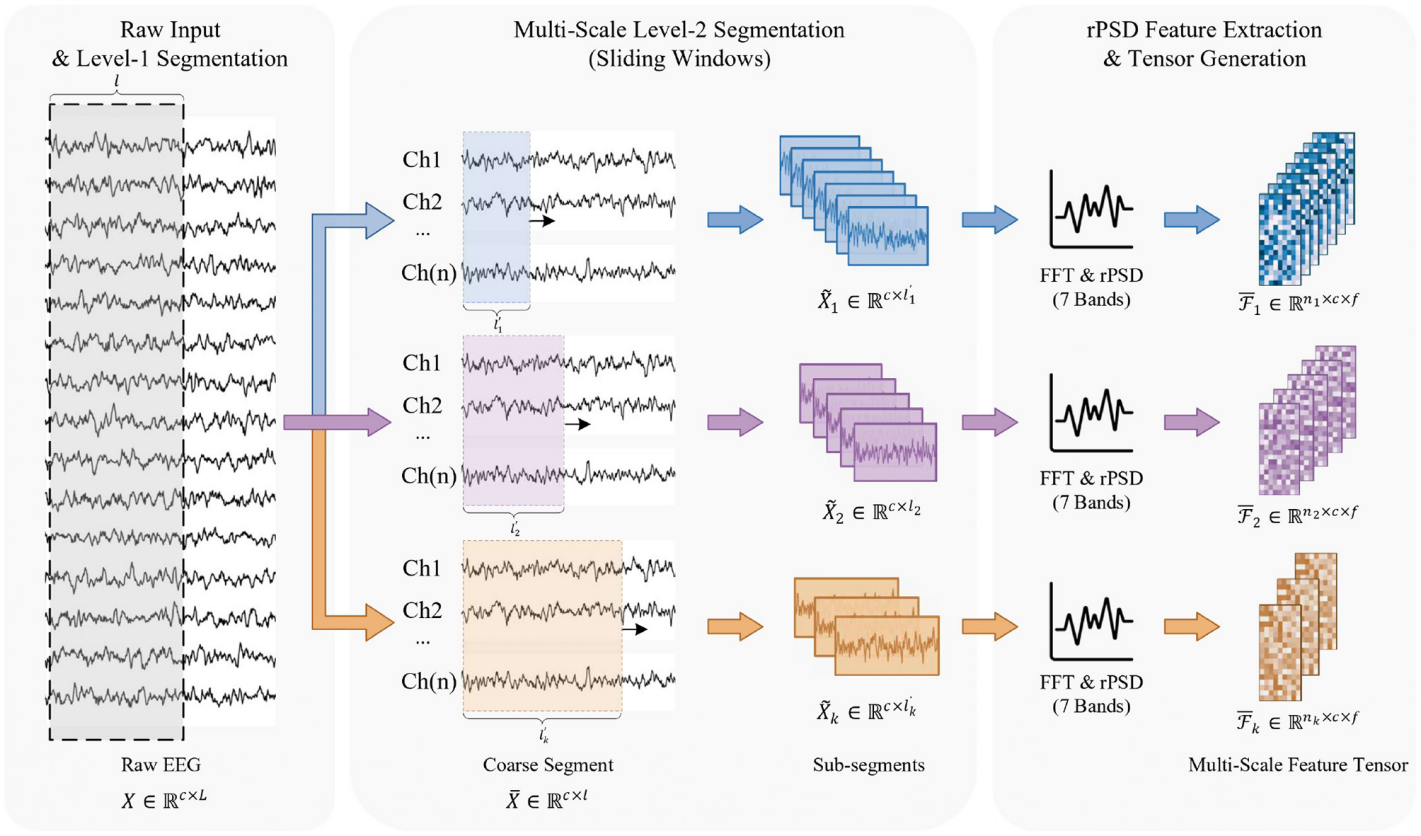
The schematic of Temporal Multi-Scale Feature Extraction. The process begins with raw EEG signals undergoing a two-level segmentation strategy. In the second level, sliding windows of varying lengths are applied to capture multi-scale temporal dynamics. Finally, rPSD features are computed for each sub-segment and stacked to generate the multi-scale feature tensors used as input for the subsequent spatial module.

To effectively analyze emotional states from EEG signals, a multi-scale feature extraction process is employed , as illustrated in Figure 2. This section details the three key stages: multi-scale temporal segmentation, frequency-domain feature extraction using relative power spectral density (rPSD), and multi-scale feature tensor generation.

#### 2.1.1 Multi-scale temporal segmentation

The raw EEG signal, denoted as *X* ∈ ℝ^*c*×*L*^ where *c* represents the number of channels and *L* indicates the total number of time samples, is processed through a two-level segmentation method to effectively capture its multi-scale temporal dynamics. In the initial stage, known as first-level segmentation, the signal *X* is segmented into larger portions $\bar{X}\in {\mathbb{R}}^{c\times l}$ by applying a sliding window of length *l* = 20*sec* = 20 * *f*_*s*_ with a hop step *s* = 4*sec* = 4 * *f*_*s*_, where *f*_*s*_ represents the sampling frequency of EEG signals, resulting in overlapping segments that encompass wider temporal contexts within the EEG data. Following this, the second-level segmentation takes each of these larger segments $\bar{X}$ and further divides them into smaller sub-segments using *k* distinct sliding windows, each characterized by specific lengths $l_{k}^{\prime }$ and hop steps $s_{k}^{\prime }$ for *k* = 1, 2, 3, …, *k*. This process yields *k* sets of sub-segments ${\tilde{X}}_{k}\in {\mathbb{R}}^{c\times l_{k}^{\prime }}$, with each set offering a unique temporal resolution of the brain activity contained within the same larger segment.

#### 2.1.2 Frequency-domain feature extraction

For each sub-segment ${\tilde{X}}_{k}$ derived from the *k* different time window lengths, spectral features are extracted by applying the Fast Fourier Transform (FFT) to each channel. The signal is decomposed into seven frequency bands: delta (1–4 Hz), theta (4–8 Hz), alpha (8–12 Hz), low beta (12–16 Hz), beta (16–20 Hz), high beta (20–28 Hz), and gamma (30–45 Hz). The relative power spectral density (rPSD) is then computed for each band using Welch's method, yielding a feature matrix $F_{k}\in {\mathbb{R}}^{c\times f}$ for each sub-segment ${\tilde{X}}_{k}$, where *f* = 7. The selection of rPSD over traditional Power Spectral Density (PSD) or Differential Entropy (DE) is predicated on its enhanced robustness against inter-subject physiological variability. Absolute metrics such as PSD and DE are highly sensitive to non-task-related factors, including individual variations in skull thickness and electrode-skin impedance, which can significantly modulate the absolute amplitude of the recorded EEG. By normalizing the power of specific frequency bands relative to the total spectral power, rPSD provides a scale-invariant representation that emphasizes the proportional distribution of neural oscillations. This approach effectively mitigates the domain shift observed in subject-independent recognition tasks and ensures that the model captures the relative shifts in rhythmic activity most characteristic of emotional transitions. These rPSD values are later used as node attributes in the graph representation.

#### 2.1.3 Multi-scale feature tensor generation

The rPSD features extracted from the previous step are organized into *k* distinct feature tensors, each corresponding to one of the temporal scales defined by the window sizes $l_{1}^{\prime },l_{2}^{\prime },\ldots ,l_{k}^{\prime }$. For each scale *k*, the resulting feature tensor is structured as $F_{k}\in {\mathbb{R}}^{b\times n_{k}\times c\times f}$, where *b* is the batch size, *n*_*k*_ is the number of segments for the *k*-th window size. This multi-scale tensor representation preserves the temporal information at different granularities and provides a comprehensive spatio-temporal characterization of the EEG signals.

### 2.2 Spatial multi-scale prior information initialization

This subsection outlines a method for initializing spatial prior information across multiple scales in EEG analysis. The approach involves three key steps: preprocessing and feature preparation to extract relevant EEG features, construction of global and local graphs to model channel interactions, and enhancement of multi-scale spatial priors to improve the representation of connectivity patterns.

#### 2.2.1 Preprocessing and feature preparation

Using the preprocessed multi-scale feature tensor $F_{k}\in {\mathbb{R}}^{b\times n_{k}\times c\times f}$, spatial graphs are constructed to represent channel interactions. To establish a consistent graph structure across the batch, we compute the average of $F_{k}$ over the batch dimension, yielding ${\bar{F}}_{k}\in {\mathbb{R}}^{n_{k}\times c\times f}$. This averaging reduces computational complexity while preserving common spatial patterns within the data. Subsequently, ${\bar{F}}_{k}$ is reshaped into a matrix $Z\in {\mathbb{R}}^{c\times \left(fn_{k}\right)}$ by flattening the sequence and feature dimensions. To adaptively combine features across frequency bands and time segments, a learnable transformation is applied:


$$\begin{array}{l} Z=ZW+V, \end{array}$$ (1)

where $W\in {\mathbb{R}}^{\left(fn_{k}\right)\times n_{k}}$ is a trainable weight matrix initialized using Xavier uniform initialization to ensure stable gradient flow during training, and $V\in {\mathbb{R}}^{c\times n_{k}}$ is a bias matrix initialized as zeros. This transformation enables the model to learn optimal feature combinations, enhancing its sensitivity to emotional patterns embedded in the EEG signals.

#### 2.2.2 Construction of global and local graphs

**Figure 3:**
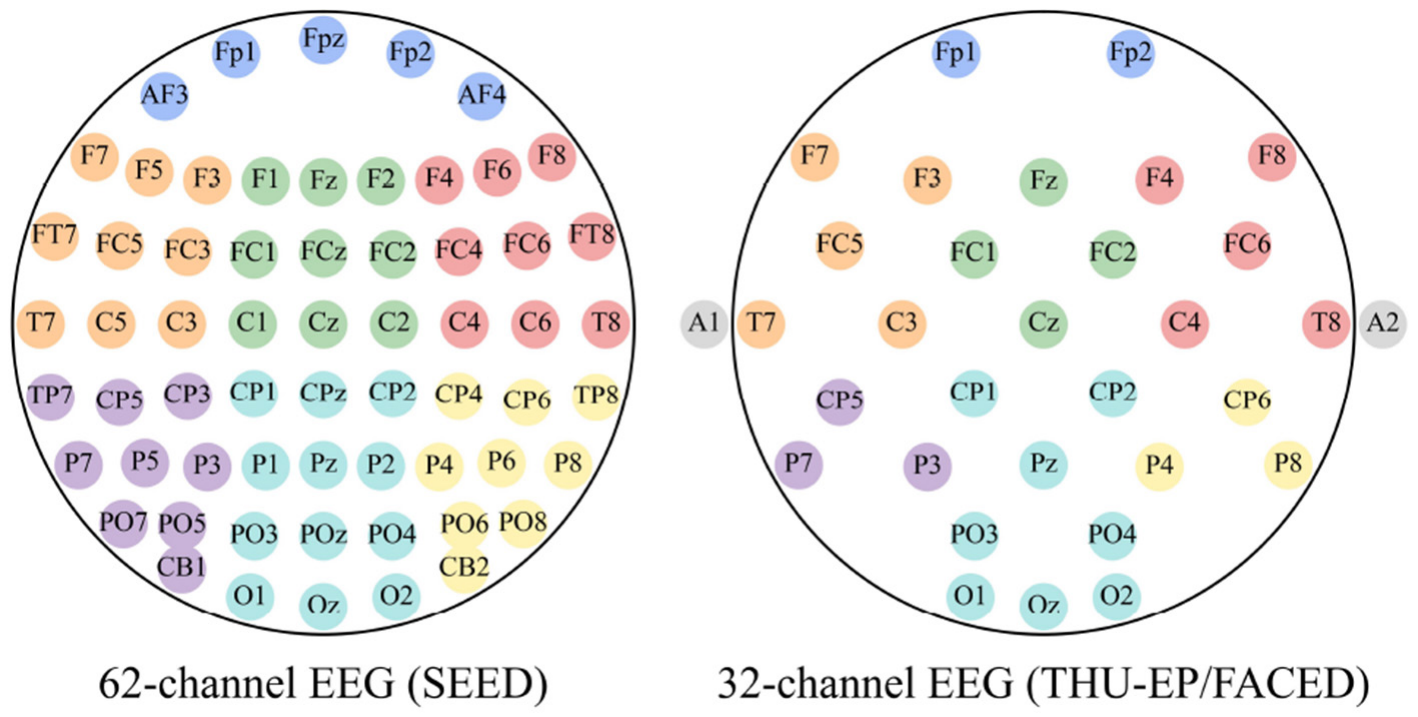
The division method of 62-channel and 32-channel EEG. The same color represents the same region.

At each scale *k*, two graphs are defined: a global graph *G*_*G, k*_ = (*U, E*_*G, k*_) and a local graph *G*_*L, k*_ = (*U, E*_*L, k*_). Both graphs share the same node set *U* = {*u*_1_, *u*_2_, …, *u*_*c*_}, where each node *u*_*i*_ corresponds to an EEG channel, and the feature vector for node *u*_*i*_, denoted ${\text{u}}_{i,k}\in {\mathbb{R}}^{n_{k}}$, is extracted directly from *Z*. The global adjacency matrix $W_{G,k}\in {\mathbb{R}}^{c\times c}$ is constructed using a hybrid metric that integrates the Pearson Correlation Coefficient (PCC) and Manhattan Distance (MD) to eliminate weak or noisy connections while retaining meaningful spatial relationships. The PCC, κ_*i, j, k*_, is calculated after normalizing the feature vectors by subtracting their mean and dividing by their standard deviation, with a small constant (1*e* − 6) added to the denominator to avoid division by zero in cases of constant features. The MD is computed as *d*_*i, j, k*_ = ||**u**_*i, k*_ − **u**_*j, k*_||_1_, capturing the absolute differences between feature vectors. The weights in *W*_*G, k*_ are then defined as:


$$\begin{array}{l} w_{ij}^{G,k}=\{\begin{array}{ll} \exp\left(-\frac{\|u_{i,k}-u_{j,k}{\|}_{2}^{2}}{2\sigma^{2}}\right), & \begin{array}{l} \text{if }\kappa_{i,j,k}\geq \kappa_{\theta }\\ \text{and }d_{i,j,k}\leq d_{\theta }, \end{array}\\ 0, & \text{otherwise}, \end{array} \end{array}$$ (2)

where σ is the Gaussian kernel bandwidth, adaptively set to (μ_*d*_ + σ_*d*_)/2–the average of the mean (μ_*d*_) and standard deviation (σ_*d*_) of Euclidean distances across all node pairs–unless specified otherwise. The thresholds κ_θ_ and *d*_θ_ are set to the 75th percentile of PCC values and the 25th percentile of MD values, respectively, ensuring data-driven robustness without requiring manual tuning. In contrast, the local adjacency matrix $W_{L,k}\in {\mathbb{R}}^{c\times c}$ restricts connectivity to channels within predefined scalp regions (see Figure 3), defined as:


$$\begin{array}{l} w_{ij}^{L,k}=\{\begin{array}{ll} w_{ij}^{G,k}, & \text{if }u_{i}\text{ and }u_{j}\text{ are in the same region},\\ 0, & \text{otherwise}. \end{array} \end{array}$$ (3)

This dual-graph strategy effectively encapsulates both extensive inter-channel dependencies and localized interactions, forming a comprehensive spatial prior for EEG analysis.

#### 2.2.3 Multi-scale spatial prior enhancement

To enhance the multi-scale spatial priors, the global and local adjacency matrices *W*_*G, k*_ and *W*_*L, k*_ at each scale *k* are duplicated and stacked along a new dimension, resulting in tensors $G_{G,k}=\left[\begin{array}{c} W_{G,k}\\ W_{G,k} \end{array}\right]$and $G_{L,k}=\left[\begin{array}{c} W_{L,k}\\ W_{L,k} \end{array}\right]$, both of shape (2, *c, c*). Although the duplicated graphs are identical in this initial setup, this structure provides flexibility for subsequent layers to apply distinct transformations or attention mechanisms, potentially enriching the representation of spatial relationships.

### 2.3 Spatiotemporal feature adaptive fusion

This subsection introduces the spatiotemporal feature adaptive fusion module, which captures dynamic spatial relationships among EEG channels for emotion analysis by integrating multi-depth Graph Convolutional Networks (GCNs) and temporal fusion via token embeddings.

#### 2.3.1 Adaptive graph encoding with multi-depth GCNs

The core of the spatiotemporal feature adaptive fusion module leverages four distinct Graph Encoders, each implemented using ChebyNet (Defferrard et al., 2016), a variant of GCN that employs Chebyshev polynomials to approximate spectral graph convolutions. The graph convolution operation is formally defined as:


$$\begin{array}{l} \Phi_{g}\left(F,A\right)=\sigma \left(\overset{I-1}{\underset{i=0}{\sum }}\theta^{i}T_{i}\left(\hat{L}\right)F-B\right), \end{array}$$ (4)

where *F* is the input feature matrix, *A* is the adjacency matrix, $\hat{L}$ is the normalized Laplacian, *T*_*i*_ are Chebyshev polynomials of order *I*, θ^*i*^ are learnable parameters, and σ denotes the ReLU activation.

These four Graph Encoders process the graphs in the following manner. The Shallow Global GCN ($\Phi_{g,\text{shallow}}^{G}$) applies a shallow GCN to the first global graph $G_{G,k}^{\left(1\right)}$. In contrast, the Deep Global GCN ($\Phi_{g,\text{deep}}^{G}$) employs a deeper GCN on the second global graph $G_{G,k}^{\left(2\right)}$. Similarly, the Shallow Local GCN ($\Phi_{g,\text{shallow}}^{L}$) processes the first local graph $G_{L,k}^{\left(1\right)}$ with a shallow GCN, while the Deep Local GCN ($\Phi_{g,\text{deep}}^{L}$) applies a deeper GCN to the second local graph $G_{L,k}^{\left(2\right)}$.

#### 2.3.2 Spatiotemporal fusion via token embeddings

A linear projection layer, LP(·), transforms the flattened input features $\Gamma \left(F_{k}\right)\in {\mathbb{R}}^{\left(b\cdot n_{k}\right)\times \left(c\cdot f\right)}$ into a base embedding $H_{g-\text{base},k}\in {\mathbb{R}}^{\left(b\cdot n_{k}\right)\times h}$, where *h* is the hidden dimension, providing a non-filtered representation of the input graph.

The outputs from the GCNs and the base embedding are combined separately for the global and local graphs at each scale *k*. The Global Graph Embedding is computed as:


$$\begin{array}{l} s_{G,k}=\text{mean}(H_{g-\text{base},k},\Phi_{g,\text{shallow}}^{G}({ℱ}_{k},A_{G,k}^{\left(1\right)}),\Phi_{g,\text{deep}}^{G}({ℱ}_{k},A_{G,k}^{\left(2\right)})), \end{array}$$ (5)

where $s_{G,k}\in {\mathbb{R}}^{\left(b\cdot n_{k}\right)\times h}$. Similarly, the Local Graph Embedding is defined as:


$$\begin{array}{l} s_{L,k}=\text{mean}(H_{g-\text{base},k},\Phi_{g,\text{shallow}}^{L}({ℱ}_{k},A_{L,k}^{\left(1\right)}),\Phi_{g,\text{deep}}^{L}({ℱ}_{k},A_{L,k}^{\left(2\right)})), \end{array}$$ (6)

where $s_{L,k}\in {\mathbb{R}}^{\left(b\cdot n_{k}\right)\times h}$. These embeddings are generated for each temporal scale, producing a set of global and local token embeddings {*s*_*G, k*_, *s*_*L, k*_} that encapsulate multi-view spatial representations. These tokens are subsequently passed to the MSST-Mamba, which learns temporal dependencies across scales, effectively integrating both spatial and temporal patterns present in the EEG data.

### 2.4 MSST-Mamba

The MSST-Mamba module constitutes a crucial component within a broader framework designed to capture the multi-scale spatiotemporal dynamics of EEG signals. It processes an input tensor through a stack of *m* MSSTBlock layers, followed by a final normalization step. Given an input $x_{\text{in}}\in {\mathbb{R}}^{b\times n_{k}\times h}$ reshaped from the preceding spatiotemporal feature adaptive fusion module, the module's computation is expressed as:


$$\begin{array}{l} x_{m}={\text{MSSTBlock}}_{m}\left(\text{RMSNorm}\left(x_{m-1}\right)\right)+x_{m-1}, \end{array}$$ (7)

where *m* = 1, …, *M* denotes the layer index. The final output is normalized via Root Mean Square Normalization (RMSNorm) (Zhang and Sennrich, 2019) to ensure numerical stability.

Each MSSTBlock encapsulates a MambaBlock (Gu and Dao, 2023) that performs the core temporal modeling. Following the standard architecture, the MambaBlock expands the input dimension via linear projections, splits it into a convolutional branch (using a 1D depthwise convolution and SiLU activation) and a gating branch, and processes the features through the Selective State-Space Model (SSM). The core recurrence of the selective scan is defined as:


$$\begin{array}{l} x_{t}=\exp\left(\Delta_{t}\cdot A\right)\cdot x_{t-1}+\Delta_{t}\cdot B_{t}\cdot v_{t}, \end{array}$$ (8)

$$\begin{array}{l} y_{t}=C_{t}\cdot x_{t}+D\cdot v_{t}, \end{array}$$ (9)

where *v*_*t*_ is the input slice at time *t*, and Δ_*t*_, *B*_*t*_, and *C*_*t*_ are time-varying parameters generated from the input via linear projections, enabling the model to selectively propagate or forget information. This mechanism captures both short- and long-term dependencies with linear complexity in *n*_*k*_.

### 2.5 Classifier

After processing through the MSST-Mamba module, the global and local *x*_out_ are mean-pooled along the sequence dimension and L2-normalized, then averaged to produce scale-specific representations, which are fused across all *k* scales via mean pooling to form a unified embedding $x_{\text{final}}\in {\mathbb{R}}^{b\times h}$. This embedding captures multi-scale spatiotemporal information from the EEG signals.

The final classification output, $ŷ\in {\mathbb{R}}^{b\times d_{\text{out}}}$, where *d*_out_ represents the number of emotion classes, is generated by a linear classifier applied to the unified embedding:


$$\begin{array}{l} ŷ=x_{\text{final}}W+b \end{array}$$ (10)

Here, $W\in {\mathbb{R}}^{h\times d_{\text{out}}}$ and $b\in {\mathbb{R}}^{d_{\text{out}}}$ are the learnable weights and bias, respectively. This linear layer maps the multi-scale embedding to the logit space, producing logits that can be transformed into a probability distribution over emotion classes using the softmax function during inference or training with a cross-entropy loss.

## 3 Experiment and results

### 3.1 Datasets

To assess the performance of our proposed model, we conducted comprehensive experiments utilizing five publicly available datasets: the SJTU Emotion EEG Dataset (SEED) (Zheng and Lu, 2015), the Emotion Profiles dataset (THU-EP) (Hu et al., 2022), and its expanded counterpart, the FACED dataset (Chen et al., 2023).

The SEED dataset, developed by Shanghai Jiao Tong University's BCMI laboratory, contains EEG recordings from 15 native Chinese participants (seven males, eight females; mean age: 23.27 years). These subjects watched 15 Chinese film clips, each lasting about 4 min, selected to evoke three emotional states: positive, neutral, and negative (five clips per category). Following each clip, participants rated their emotions based on valence and arousal. Brain activity was captured using a 62-channel electrode cap configured per the 10–20 system, with signals recorded at a 1,000 Hz sampling rate. The data was preprocessed with a 0.3–50 Hz bandpass filter to enhance signal quality for emotion analysis.

The THU-EP dataset includes EEG data from 80 college students (50 females, 30 males; aged 17–24, mean: 20.16 years) exposed to 28 video clips averaging 67 s each. These clips were designed to trigger nine emotions: anger, disgust, fear, sadness, amusement, joy, inspiration, tenderness, and neutral, with four clips for neutral and three for each of the others. The experiment was divided into seven blocks of four trials, with participants solving 20 arithmetic problems between blocks to reset their emotional baseline. After each clip, subjects self-reported scores for arousal, valence, familiarity, and liking. EEG signals were recorded using the NeuSen.W32 wireless system with 32 channels at a 250 Hz sampling rate, preprocessed with a 0.05–47 Hz bandpass filter, and cleaned via independent component analysis (ICA) to remove artifacts.

The FACED dataset builds on THU-EP, expanding to 123 subjects by adding 43 participants to the original 80, while retaining the same experimental framework. It employs the identical 28 video clips to elicit the nine emotions from THU-EP, following the same seven-block, four-trial structure with arithmetic tasks between blocks. Post-clip self-reports of emotional scores mirror THU-EP's methodology. EEG data was collected with the 32-channel NeuSen.W32 system at 250 Hz, and preprocessing aligns with THU-EP, using a 0.05–47 Hz bandpass filter and ICA for artifact removal. This larger dataset enhances the scope for studying EEG-based emotional responses.

### 3.2 Baseline methods

This investigation appraises the effectiveness of our methodology in EEG-based emotion recognition. We benchmark it against a suite of recognized baseline approaches, detailed hereafter:

(1) DGCNN (graph-based) (Song et al., 2020): the Dynamical Graph Convolutional Neural Network (DGCNN) dynamically discerns inter-channel EEG relationships via a trainable adjacency matrix, refined throughout the neural network's learning process. This adaptability markedly enhances the extraction of discriminative spatial features, bolstering emotion recognition precision.
(2) RGNN (graph-based) (Zhong et al., 2020): the Regularized Graph Neural Network (RGNN) leverages neuroscientific insights into brain topology to model local and global EEG channel interactions. By embedding sparsity-inducing regularization within its graph convolutions, RGNN prunes extraneous connections, thereby amplifying emotionally salient features and ensuring robust classification across diverse stimuli.
(3) PGCN (graph-based) (Jin et al., 2023): the Pyramidal Graph Convolutional Network (PGCN) constructs a triadic hierarchy–encompassing local electrode clusters, mesoscopic regions (e.g., seven lobes), and global cortex–using sparse adjacency matrices. This hierarchical synthesis mitigates over-smoothing, yielding a precise and interpretable emotional activity map.
(4) TSception (CNN-based) (Ding et al., 2022): TSception, a multi-scale convolutional architecture, integrates dynamic temporal, asymmetric spatial, and fusion layers. By concurrently extracting temporal dynamics and spatial asymmetries, it excels in discerning rapid emotional fluctuations across EEG channels.
(5) LSTM (temporal-learning) (Soleymani et al., 2016): Long Short-Term Memory (LSTM) networks , equipped with dual memory cells and gating mechanisms, process 4 Hz EEG sequences to capture long-term temporal dependencies. Such capability proves invaluable for tracking gradual emotional transitions, e.g., neutral to positive valence.
(6) TCN (temporal-learning) (Zhang et al., 2022): the Temporal Convolutional Network (TCN) employs adjustable dilated convolutions, augmented by visual-to-EEG distillation, to encapsulate extended temporal patterns, outperforming LSTM in multimodal regression tasks.
(7) BiDANN (adversarial-based) (Li et al., 2021): the Bi-Hemisphere Domain Adversarial Neural Network (BiDANN) deploys dual-hemisphere LSTM extractors feeding three discriminators, interlinked via Gradient Reversal Layers. This adversarial domain alignment, preserving hemispheric distinctions, ensures robust cross-subject generalization.
(8) DMATN (adversarial-based) (Wang et al., 2021): the Deep Multi-Source Adaptation Transfer Network (DMATN) synthesizes multi-source EEG through attention-weighted fusion and an adversarial classifier. By harmonizing diverse inputs, it achieves consistent cross-subject performance.
(9) EmT (Graph-Transformer-Based) (Ding et al., 2025): the Emotion Transformer (EmT), a leading method in graph-transformer-based EEG emotion recognition, leverages a graph-transformer architecture to model spatiotemporal dynamics. By converting signals into temporal graphs, its residual multi-view pyramid GCN (RMPG) captures diverse spatial patterns of emotional cognition, while the temporal contextual transformer (TCT) excels at learning long-term dependencies, achieving superior cross-subject generalization in classification and regression tasks.

### 3.3 Experimental protocol

In this study, we adopt a training strategy consistent with the approach in EmT to implement a rigorous k-fold subject-independent evaluation framework, ensuring effective generalization to unseen individuals across tailored cross-validation strategies for the SEED, THU-EP, and FACED datasets. This protocol is more demanding than standard k-fold partitioning as it strictly prevents potential data leakage between training and testing sets. For the SEED dataset, which includes data from fifteen experimental subjects, we employ a 15-fold leave-one-subject-out (LOSO) cross-validation approach, where in each fold, data from one subject are set aside as the test set, and the remaining fourteen subjects' data are pooled and split randomly into training and validation sets at an 8:2 ratio—80% for training and 20% for validation. This subject-independent validation ensures that the model learns generalized neural signatures rather than memorizing individual-specific artifacts, providing a more robust measure of performance in real-world clinical scenarios. As previously noted, we sliced the SEED dataset into time windows of varying lengths to serve as input, resulting in data segments of different sizes. To accommodate this variability, we created multiple dataloaders to feed the network, ensuring consistent labeling across all segments. For the THU-EP and FACED datasets, we use a 10-fold leave-n-subject-out cross-validation strategy, with *n* set to 8 for THU-EP and 12 for FACED; in each fold, data from *n* subjects form the test set, while the remaining subjects' data are divided so that 90% go to training and 10% to validation. Across all three datasets, we classify emotions binarily into positive and negative categories, and for THU-EP and FACED, this involves converting valence scores into high and low categories using a 3.0 threshold. The model is trained on the training set, using the validation set to tune hyperparameters and avoid overfitting, and its performance is assessed on the test set; this process repeats for each fold, with final performance metrics averaged across all iterations.

### 3.4 Parameter settings

The training configuration of the proposed MSGM model is detailed in Table 1. The model employs cross-entropy loss for optimization, guided by the AdamW optimizer with an initial learning rate of 3 × 10^−4^. To address overfitting, label smoothing (0.1) and dropout (0.25) are applied. A batch size of 32 is used across all datasets, with training epochs set to 20 for SEED and 30 for THU-EP and FACED, incorporating an early stopping mechanism with a patience of 5. The model with the highest validation accuracy is selected for testing.

**Table 1:** Training hyperparameters of the MSGM model.

**Hyperparameters**	**Value**
Loss function	Cross-entropy
Optimizer	AdamW
Initial learning rate	3 × 10^−4^
Label smoothing	0.1
Dropout rate	0.25
Batch size	32
Training epochs (SEED)	20
Training epochs (THU-EP, FACED)	30
Early stopping patience	5

The MSST-Mamba architecture, summarized in Table 2, leverages two Chebyshev graph encoders with layers [1, 2] to enhance graph processing and handle complex relationships effectively. It employs an embedding dimension *h* = 32 and a convolutional kernel size of 4 to capture local temporal patterns efficiently. A single MSST-Mamba layer is adopted for feature extraction and spatiotemporal processing across datasets, achieving high accuracy while maintaining computational efficiency. The selective state-space model (SSM) operates with a dynamically computed *dt*_rank_ = ⌈*h*/16⌉ and a state dimension *d*_state_ = 16, optimizing spatiotemporal modeling.

**Table 2:** Architectural hyperparameters of the MSGM model.

**Hyperparameters**	**Value**
GCN layers	[1, 2]
Embedding dimension (*h*)	32
MSST-Mamba layers	1
*dt*_rank_	⌈*h*/16⌉
*d*_state_	16

Hardware configurations are presented in Table 3. Training and testing leverage an NVIDIA GeForce RTX 3070Ti (8 GB GDDR6), enabling rapid optimization of the model's parameters. For real-world deployment, the NVIDIA Jetson Xavier NX, featuring a 6-core Carmel ARM v8.2 CPU and a Volta GPU with 48 Tensor Cores (up to 21 TOPS, INT8), offers low-power (10–20 W) and high-efficiency inference, supported by 8 GB LPDDR4x memory and 51.2 GB/s bandwidth, ideal for edge computing applications.

**Table 3:** Hardware specifications for training and deployment.

**Property**	**GeForce RTX 3070Ti**	**Jetson Xavier NX**
GPU	NVIDIA GeForce RTX 3070Ti	NVIDIA Volta
CPU	Core i7-8700K	Carmel Arm v8.2
RAM	64 GB	8 GB
Power usage	240 W	10 W/15 W/20 W
Purpose	Training and testing	Real-world deployment

## 4 Numerical results

### 4.1 Emotion recognition performance

The experimental results are presented in Table 4, which evaluates the performance of various methods for generalized emotion classification across three datasets–SEED, THU-EP, and FACED–using accuracy (ACC %) and F1 score (F1 %) as metrics. Across all datasets, the observed consistency between Accuracy and F1-score suggests that MSGM maintains a balanced classification performance, effectively mitigating bias toward either positive or negative emotional states. On the SEED dataset, our proposed method achieves an outstanding accuracy of 83.43% and an F1 score of 85.03%, outperforming all other approaches. This performance indicates that the model successfully captures generalized spatiotemporal features that remain robust across different emotional categories.

**Table 4:** The accuracies and F1 scores (mean ± SD) on the SEED, THU-EP, and FACED datasets.

**METHOD**	**SEED**		**THU-EP**		**FACED**	
	**ACC (%)**	**F1 (%)**	**ACC (%)**	**F1 (%)**	**ACC (%)**	**F1 (%)**
KNN	49.26 ± 14.89	48.89 ± 24.91	23.45 ± 4.82	30.93 ± 4.85	22.71 ± 5.34	31.89 ± 4.37
SVM	51.68 ± 17.86	50.31 ± 28.76	24.72 ± 5.91	33.28 ± 8.70	25.58 ± 7.43	32.32 ± 9.23
DGCNN	72.48 ± 14.89	61.95 ± 13.14	56.71 ± 3.37	64.74 ± 5.22	56.26 ± 4.55	69.78 ± 4.36
RGNN	79.07 ± 14.82	80.26 ± 13.34	57.23 ± 3.07	69.51 ± 5.49	58.71 ± 5.05	72.27 ± 72.16
DMATN	77.29 ± 15.46	77.16 ± 16.32	60.34 ± 5.41	69.22 ± 6.38	61.41 ± 4.91	68.31 ± 6.87
TSception	66.22 ± 18.11	62.18 ± 28.34	59.18 ± 5.93	70.72 ± 6.01	61.92 ± 8.81	70.26 ± 23.76
TCN	76.54 ± 14.08	73.77 ± 21.96	57.79 ± 3.13	67.71 ± 3.12	55.26 ± 3.56	67.31 ± 3.53
PGCN	75.87 ± 18.36	74.08 ± 19.01	56.92 ± 4.31	65.14 ± 9.06	55.77 ± 7.81	66.54 ± 8.52
LSTM	73.39 ± 15.88	67.05 ± 27.47	55.83 ± 3.52	62.69 ± 6.22	56.84 ± 6.31	70.07 ± 6.44
BiDANN	79.39 ± 16.45	77.65 ± 15.92	61.44 ± 5.51	69.71 ± 6.87	**63.36** **±7.01**	73.82 ± 6.36
EmT^*^	80.20 ± 11.50	82.10 ± 9.30	59.50 ± 4.70	72.40 ± 4.40	60.80 ± 6.50	74.00 ± 5.80
**MSGM (Proposed)**	**83.43** **±11.42**	**85.03** **±9.09**	**62.39** **±3.13**	**73.28** **±4.39**	63.17 ± 3.62	**76.01** **±3.74**

The best results are highlighted in bold and the next best are marked using underlines. ^*^Indicates the model's results are sourced from the cited paper. Models without ^*^ reflect local experimental results.

To rigorously evaluate the superiority of MSGM, we conducted statistical significance testing using Welch's *t*-test, as detailed in Table 5. MSGM exhibits statistically significant improvements over most baseline models, particularly traditional architectures. On the SEED dataset, MSGM significantly outperforms DGCNN (*p* = 0.032) and TSception (*p* = 0.005) with large effect sizes (Cohen's *d*>0.8). Although the margin over EmT and BiDANN does not consistently reach the *p* < 0.05 threshold due to the high inter-subject variance, the positive Cohen's *d* values across almost all comparisons indicate a consistent advantage in modeling efficiency.

**Table 5:** Statistical significance analysis of MSGM compared to key baseline methods using Welch's *t*-test.

**Dataset**	**Baseline method**	**Accuracy (%)**			*t*	*p* **-value**	*d*
		**MSGM**	**Base**.	Δ			
SEED	EmT	83.43	80.20	+3.23	0.77	0.447	0.28
	BiDANN	83.43	79.39	+4.04	0.78	0.442	0.29
	DMATN	83.43	77.29	+6.14	1.24	0.227	0.45
	RGNN	83.43	79.07	+4.36	0.90	0.375	0.33
	DGCNN	83.43	72.48	+10.95	2.26	0.032^*^	0.83
THU-EP	EmT	62.39	59.50	+2.89	1.62	0.126	0.72
	BiDANN	62.39	61.44	+0.95	0.47	0.643	0.21
	DMATN	62.39	60.34	+2.05	1.04	0.317	0.46
	RGNN	62.39	57.23	+5.16	3.72	0.002^**^	1.66
	DGCNN	62.39	56.71	+5.68	3.91	0.001^**^	1.75
FACED	EmT	63.17	60.80	+2.37	1.01	0.331	0.45
	BiDANN	63.17	63.36	–0.19	–0.08	0.940	–0.03
	DMATN	63.17	61.41	+1.76	0.91	0.375	0.41
	RGNN	63.17	58.71	+4.46	2.27	0.037^*^	1.02
	DGCNN	63.17	56.26	+6.91	3.76	0.002^**^	1.68

Δ denotes the improvement in accuracy. Cohen's *d* represents the effect size.

^*^p < 0.05, ^**^p < 0.01.

Cohen's *d*: |*d*| < 0.2 negligible, 0.2–0.5 small, 0.5–0.8 medium, >0.8 large.

On the FACED dataset, BiDANN achieves a slightly higher accuracy of 63.36% compared to our method's 63.17%. However, our method outperforms BiDANN in terms of F1 score, achieving 76.01% against BiDANN's 73.82%, establishing our model as the top performer in this metric. This marginal deficit in accuracy can be attributed to the inherent characteristics of the FACED dataset, which comprises a large cohort of 123 subjects with significant inter-subject variability. BiDANN explicitly incorporates a domain adversarial mechanism designed to align feature distributions across disparate subjects, effectively mitigating domain shifts. In contrast, MSGM prioritizes the extraction of complex spatiotemporal dynamics through the Mamba architecture without employing explicit domain adaptation components. Consequently, when scaling to datasets with extensive subject diversity, the distributional shifts may slightly impact the decision boundary of MSGM. Nevertheless, our performance remains statistically comparable to BiDANN (*p* = 0.940), and the superior F1 score suggests that MSGM maintains high robustness and effectively balances precision and recall even in the presence of substantial individual differences.

Beyond aggregate metrics like mean accuracy, the standard deviation (SD) reported in Table 4 offers critical insights into model stability against inter-subject variability. A closer examination of the leave-one-subject-out cross-validation results reveals that the performance variance is largely driven by a minority of subjects who exhibit significantly lower classification rates compared to the group average. This phenomenon is attributable to inherent subject-specific factors, such as physiological variations (e.g., skull thickness affecting signal quality) or fluctuating levels of emotional immersion during stimuli presentation. Notably, despite the presence of these “hard-to-classify” subjects, MSGM maintains the lowest standard deviation (±11.42%) on the SEED dataset compared to leading baselines like BiDANN (±16.45%) and DGCNN (±14.89%). This indicates that while individual disparities exist, the proposed MSST-Mamba module, constrained by neuroanatomical spatial priors, effectively extracts generalized features, preventing the model's performance from collapsing on difficult subjects and ensuring consistent predictions across the cohort.

### 4.2 Ablation study on component modules

To evaluate the contributions of the temporal multi-scale feature extraction, spatial multi-scale prior information initialization, spatiotemporal feature adaptive fusion, and MSST-Mamba and classifier modules, we conducted an ablation analysis by systematically removing each component individually and assessing its impact on classification performance. This included omitting the temporal multi-scale feature extraction (w/o Temporal Multi-Scale), spatial multi-scale prior information initialization (w/o Spatial Multi-Scale), spatiotemporal feature adaptive fusion (w/o Spatiotemporal Fusion), and MSST-Mamba (w/o MSST-Mamba), as well as replacing multi-depth GCNs with a single layer (w Single GCN), to measure each component's effect. The results are detailed in Table 6.

**Table 6:** Generalized emotion classification results of ablation studies on the SEED and THU-EP datasets (%).

**Method**	**SEED**		**THU-EP**	
	**ACC (%)**	**F1 (%)**	**ACC (%)**	**F1 (%)**
w/o Temporal Multi-Scale	80.04	80.14	59.55	72.13
w/o Spatial Multi-Scale	82.37	82.57	61.82	72.02
w Single GCN	81.92	81.50	62.01	70.68
w/o Spatiotemporal fusion	79.75	80.23	59.17	66.10
w/o MSST-Mamba	79.53	77.93	57.92	65.04
MSGM (Proposed)	**83.43**	**85.03**	**62.39**	**73.28**

The best results are highlighted in bold.

The removal of the MSST-Mamba and classifier module results in the most significant performance decline, with accuracy decreasing by 3.90% on the SEED dataset and 4.47% on the THU-EP dataset, alongside F1 score drops of 7.10 and 8.24%, respectively. This underscores its critical role in processing and integrating multi-scale spatiotemporal features effectively. Excluding the spatiotemporal feature adaptive fusion module also leads to substantial reductions, with accuracy dropping by 3.68% on SEED and 3.22% on THU-EP, highlighting its importance in unifying temporal and spatial information.

The absence of the temporal multi-scale feature extraction module decreases accuracy by 3.39% on SEED and 2.84% on THU-EP, indicating its value in capturing diverse temporal dynamics. Removing the spatial multi-scale prior information initialization module results in smaller but notable declines of 1.06% on SEED and 0.57% on THU-EP, suggesting its contribution to initializing robust spatial representations, though its impact is less pronounced than other modules. Additionally, using a single GCN instead of multiple GCN layers reduces accuracy by 1.51% on SEED and 0.38% on THU-EP, demonstrating that multi-layer GCNs more effectively capture spatial information.

### 4.3 Performance and sensitive analysis of hyperparameters

#### 4.3.1 Impact of EEG feature types

**Figure 4:**
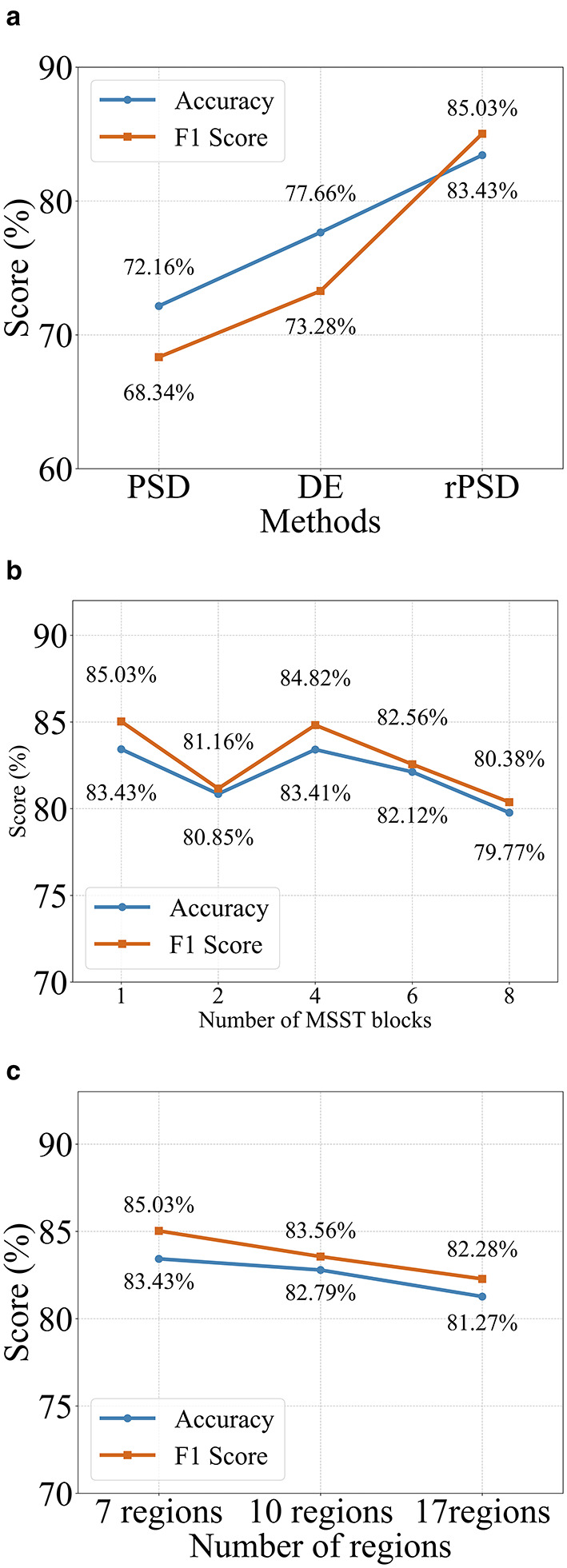
**(a)** Effect of feature types on emotion classification performances using SEED. **(b)** Effect of the number of MSST blocks on emotion classification using SEED. **(c)** Effect of different brain region partitioning methods on emotion recognition using SEED.

As illustrated in Figure 4a, which compares the accuracy and F1 scores of Power Spectral Density (PSD), Differential Entropy (DE), and relative Power Spectral Density (rPSD) features for emotion classification on the SEED dataset. Specifically, rPSD achieved an accuracy of 83.43% and an F1 score of 85.03%, surpassing DE by 5.77 percentage points in accuracy and 11.75 percentage points in F1 score. Compared to PSD, rPSD exhibited even greater improvements, with an accuracy increase of 11.27 percentage points and an F1 score increase of 16.69 percentage points. These findings demonstrate that rPSD is a superior feature in our model for EEG-based emotion classification tasks compared to both DE and PSD.

#### 4.3.2 Influence of the number of MSST-Mamba blocks

The effect of varying the number of MSST-Mamba Blocks on the emotion classification performance is illustrated in Figure 4b. The analysis considered block counts of 1, 2, 4, 6, and 8, with corresponding impacts on accuracy and F1 score. With a single block, the model achieved an accuracy of 83.43% and an F1 score of 85.03%. Increasing to 2 blocks led to a decrease in performance, with accuracy dropping to 80.85% and F1 score to 81.16%. A slight recovery was observed with 4 blocks, where accuracy reached 83.41% and F1 score 84.82%, nearly matching the single-block performance. Further increasing the block count to 6 resulted in a decline, with accuracy at 82.12% and F1 score at 82.56%, and this downward trend persisted with 8 blocks, where accuracy and F1 score further decreased to 79.77 and 80.38%, respectively. This pattern suggests that a single block achieves the best performance, with additional blocks leading to fluctuations and an overall decline at higher counts.

#### 4.3.3 Effect of prior information on brain region partitioning

**Figure 5:**
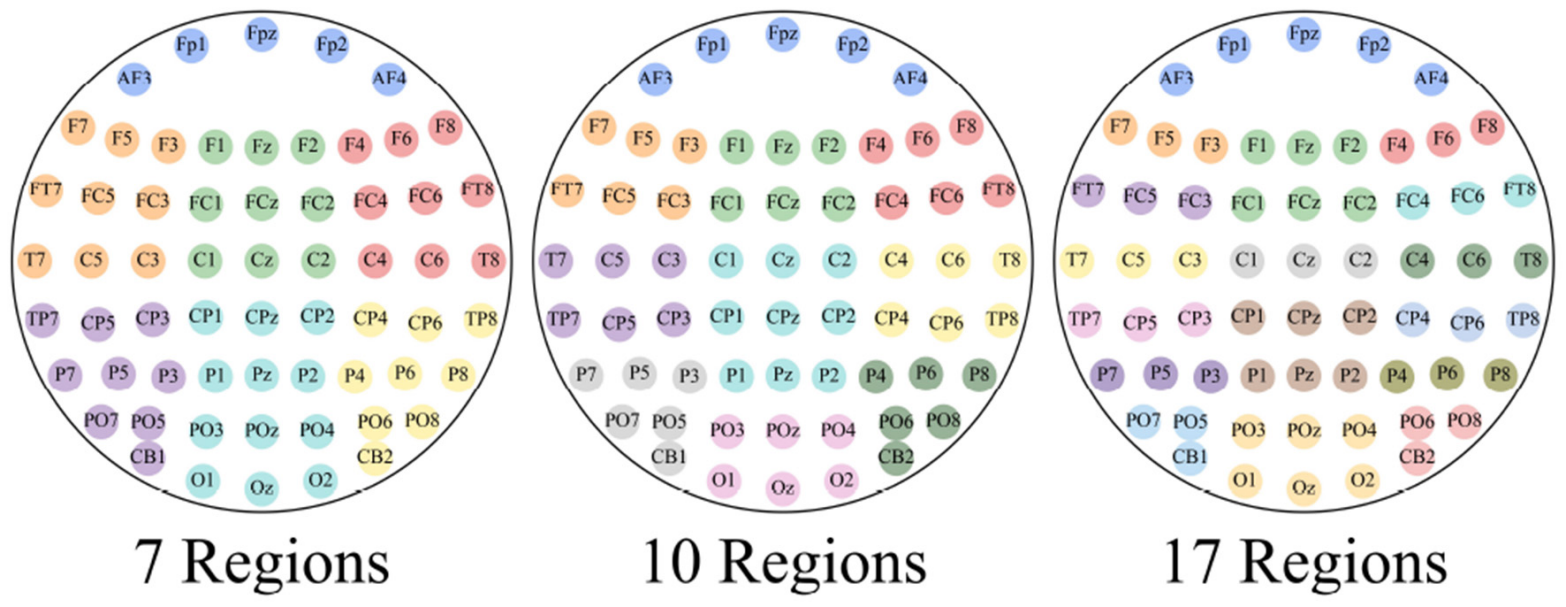
Three methods for dividing 62 EEG channels into different regions, comprising 7, 10, and 17 regions respectively.

The human brain comprises multiple functional regions, each contributing uniquely to emotional processing (Alarcao and Fonseca, 2017). The way these regions are partitioned into subgraphs can influence the structure of the EEG data representation and, consequently, the model's performance (Song T. et al., 2023). To explore this, we conducted experiments on the SEED dataset by dividing the 62 EEG channels into 7, 10, and 17 regions, as shown in Figure 5. Our results indicate (see Figure 4c) that the 7-region partitioning yields the highest accuracy (83.43%) and F1 score (85.03%), followed by the 10-region partitioning with an accuracy of 82.79% and an F1 score of 83.56%, while the 17-region partitioning produces the lowest accuracy (81.27%) and F1 score (82.28%). These findings suggest that the 7-region scheme may strike an effective balance between capturing essential functional patterns and maintaining a manageable level of complexity for the model. In contrast, the finer 17-region partitioning might overly fragment the data, diluting key inter-regional relationships, while the 10-region approach, despite performing better than 17 regions, may still not align as optimally with the underlying functional organization as the 7-region configuration.

### 4.4 Performance on edge devices

**Figure 6:**
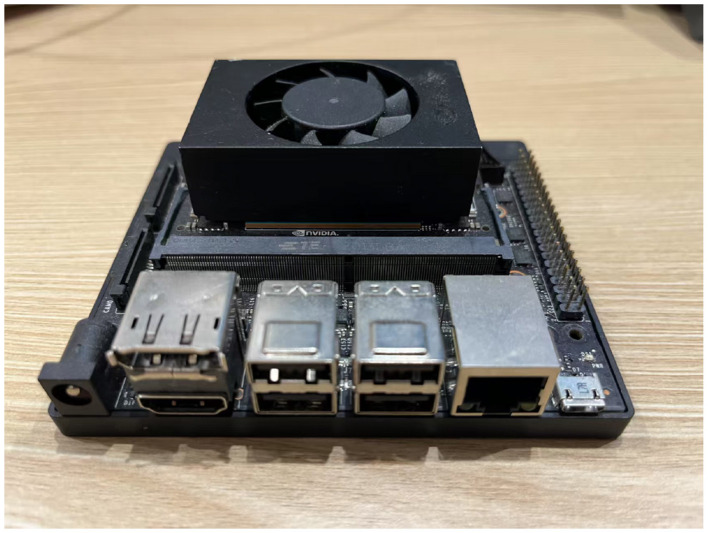
NVIDIA Jetson Xavier NX.

The MSGM model, deployed on the NVIDIA Jetson Xavier NX edge computing platform, as shown in Figure 6, exhibits efficient performance on the SEED dataset. To enable deployment on this platform, we replaced the Mamba core component in the MSST-Mamba module with Mamba-minimal(https://github.com/johnma2006/mamba-minimal), a lightweight implementation of Mamba, since the PyTorch version on the edge device does not support the official Mamba library. This substitution preserves the model's input-output functionality but results in lower runtime efficiency compared to the official Mamba implementation. With this configuration, the model utilizes 349,218 parameters and achieves an inference time of 151.0 ms, maintaining millisecond-level inference and demonstrating robust real-time processing capabilities. This efficiency underscores its suitability for edge device applications requiring rapid data handling.

### 4.5 Comparison with EmT

**Figure 7:**
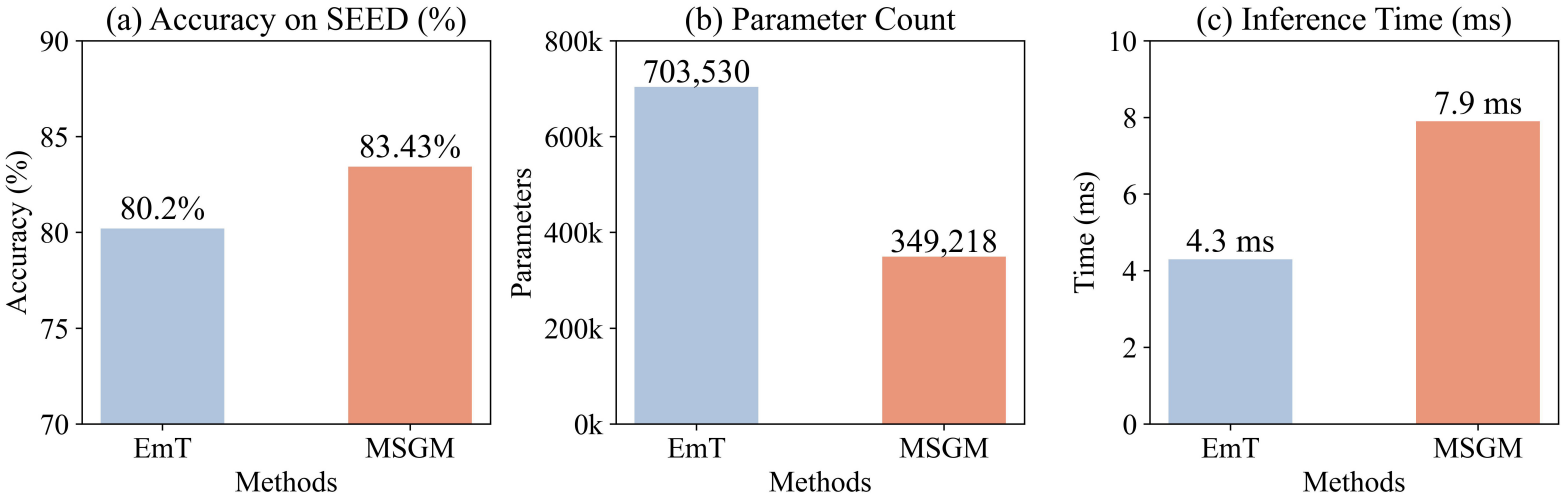
Performance comparison between the proposed MSGM and EmT. **(a)** Comparison of classification accuracy on the SEED dataset. **(b)** Comparison of total parameter counts. **(c)** Comparison of inference time on the RTX 3070Ti platform.

In this section, we compare our MSGM model with EmT, a leading method in graph-transformer-based EEG emotion recognition. Both models adopt a graph-Transformer/Mamba-based architecture to process spatial-temporal patterns in EEG signals. EmT incorporates an 8-layer TCT module, while MSGM employs a single-layer MSST-Mamba module. We evaluate their performance in terms of accuracy, parameter count, and inference time, as visually summarized in Figure 7. To ensure a fair comparison, this evaluation was conducted on the GeForce RTX 3070Ti platform (see Table 3), rather than on edge devices, allowing both MSGM and EmT to run in a consistent environment without the influence of Mamba-minimal, which was used for edge deployment.

MSGM, with its single-layer MSST-Mamba, achieves superior accuracy and F1 scores compared to EmT, despite using only 349,218 parameters–approximately half of EmT's 703,530. This highlights MSGM's efficiency, as its linear-complexity MSST-Mamba outperforms the quadratic-complexity TCT module with a simpler structure. The reduced parameter count underscores MSGM's suitability for resource-constrained settings, such as edge devices.

In terms of inference time, MSGM records 7.9 ms, slightly higher than EmT's 4.3 ms. This minor gap arises from MSGM's multi-scale architecture, which limits full parallelization. Nevertheless, both models maintain millisecond-level inference, ensuring negligible impact on real-time applications.

### 4.6 Visualization

**Figure 8:**
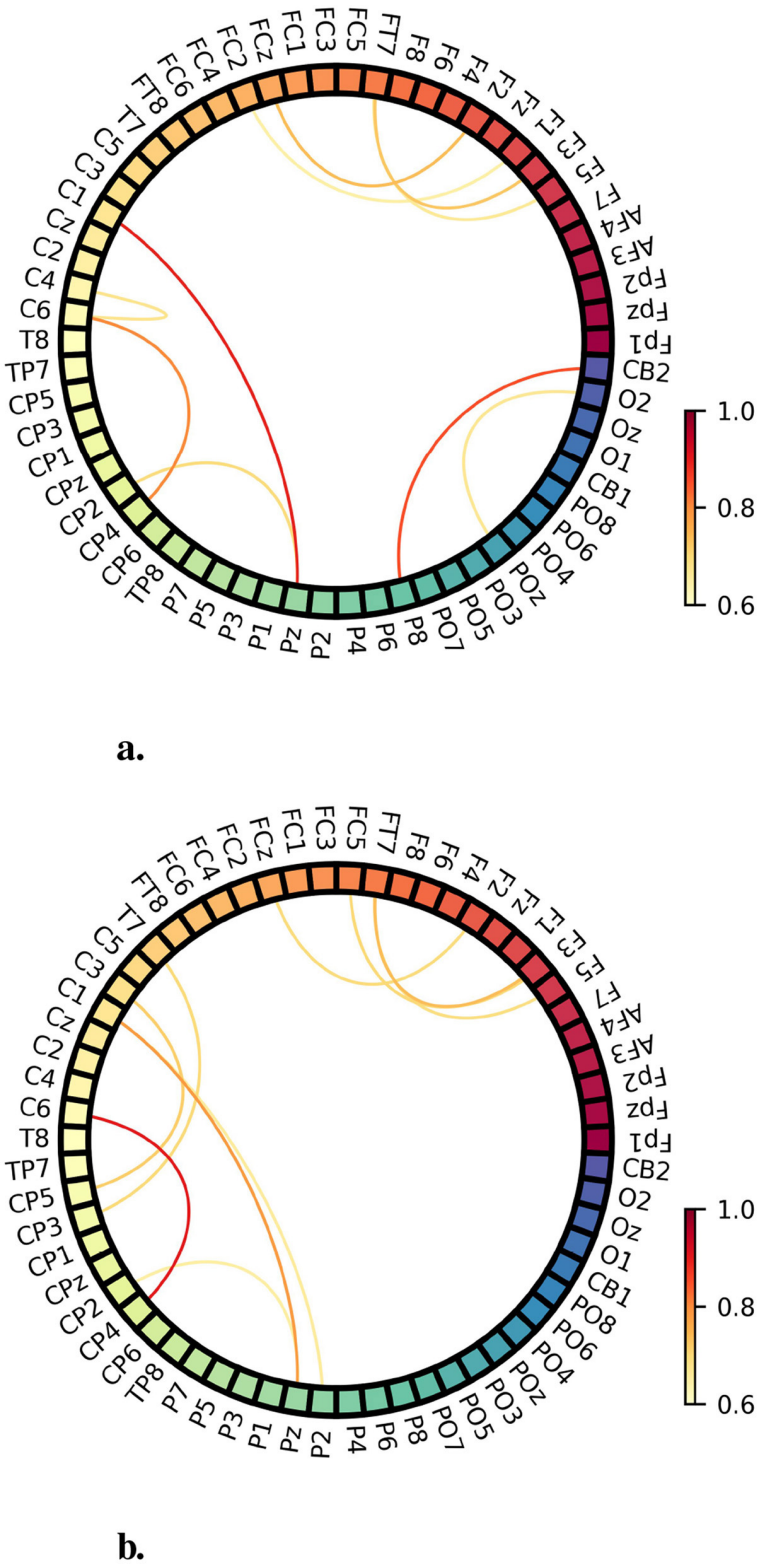
**(a)** Connectivity between the electrodes of the initial Local Graph. **(b)** Connectivity between the electrodes of the trained Local Graph.

Figure 8 presents two diagrams that illustrate the connectivity between different electrodes, derived from the initial and learned perspectives, respectively. We utilized a Local Graph derived from the Global Graph as the representation, which effectively reflects the model's learning outcomes. These results offer empirical evidence that MSGM effectively captures and retains spatial dependencies without explicitly utilizing vector-valued neurons. By encoding non-Euclidean relationships into the selective state-space, the model ensures that the functional organization of the brain informs the final classification. In the initial connectivity map (Figure 8a), the strongest connections are observed between electrodes such as C1-Pz, FC2-FPz, and C6-CP4. These connections primarily involve the central and parietal regions, with some involvement of the frontal areas (Wan et al., 2021), suggesting a baseline interaction that may reflect general neural communication prior to task-specific learning.

In contrast, the learned connectivity map (Figure 8b) reveals a more refined set of connections, with the strongest links being C6-CP4 and C1-Pz. These retained and strengthened connections continue to emphasize interactions within the central and parietal regions, which are known to play critical roles in sensory integration and spatial processing. The persistence of these specific connections suggests that the model has prioritized and enhanced these pathways, likely due to their relevance to the task at hand (Al-Qazzaz et al., 2019). Additionally, the color intensity, ranging from 0.6 to 1.0, highlights the varying strengths of these learned connections, with warmer colors indicating stronger interactions. The connectivity patterns observed in Figure 8 demonstrate the model's ability to refine and focus on key electrode relationships, transitioning from a broader initial state to a more targeted, task-driven network. This evolution confirms the model's effectiveness in capturing and prioritizing critical neural relationships without losing spatial consistency during the classification process.

## 5 Conclusion

In this paper, we propose the Multi-Scale Spatiotemporal Graph Mamba (MSGM), a novel framework for EEG-based emotion recognition that integrates temporal multi-scale feature extraction, spatial multi-scale prior information initialization, spatiotemporal feature adaptive fusion, and the MSST-Mamba module. Unlike traditional CNN or Transformer-based approaches that struggle with the trade-off between modeling complexity and computational cost, MSGM uniquely leverages the linear complexity of State Space Models. By capturing short-term emotional continuity and long-term evolutionary trends through multi-scale temporal analysis, alongside hierarchical spatial connectivity via bimodal graph modeling, MSGM addresses critical gaps in prior methodologies. Extensive experiments on the SEED, THU-EP, and FACED datasets demonstrate its superior performance over baseline methods, validated through rigorous subject-independent evaluation. The model achieves millisecond-level inference speed on edge devices like the NVIDIA Jetson Xavier NX, underscoring its practical applicability in clinical and consumer settings, while its neuroanatomical grounding enhances interpretability of the brain's distributed emotional dynamics. Despite these advancements, the accuracy has not yet surpassed 95%, a limitation primarily attributed to two intrinsic factors. First, the non-stationary nature of EEG signals and the profound inter-subject variability create significant domain shifts that are difficult to fully eliminate, even with robust neuroanatomical priors. Second, the ground truth labels rely on subjective self-assessments, introducing inherent label noise that imposes a theoretical ceiling on classification precision. Consequently, while MSGM sets a new benchmark, bridging the gap to optimal accuracy requires addressing these fundamental physiological and psychological variabilities. Looking ahead, future developments in EEG-based emotion recognition will focus on three key directions. First, integrating multimodal physiological signals, such as ECG or eye-tracking data, to enrich emotional context and improve robustness against noise. Second, we aim to optimize the architecture for specific deployment scenarios, particularly in wearable EEG systems for continuous mental state tracking. Third, we plan to explore real-time adaptive learning to dynamically adjust to individual neurophysiological profiles. These advancements will facilitate seamless integration with mobile health monitoring platforms, thereby advancing both precision and accessibility in real-world applications.

## Data Availability

The original contributions presented in the study are included in the article/supplementary material, further inquiries can be directed to the corresponding author.
